# Sequential Ras/MAPK and PI3K/AKT/mTOR pathways recruitment drives basal extrusion in the prostate-like gland of *Drosophila*

**DOI:** 10.1038/s41467-020-16123-w

**Published:** 2020-05-08

**Authors:** Amandine Rambur, Corinne Lours-Calet, Claude Beaudoin, Julio Buñay, Marine Vialat, Vincent Mirouse, Amalia Trousson, Yoan Renaud, Jean-Marc A. Lobaccaro, Silvère Baron, Laurent Morel, Cyrille de Joussineau

**Affiliations:** 1 Université Clermont Auvergne, GReD, CNRS UMR 6293, INSERM U1103, 28 place Henri Dunant, BP38, 63001 Clermont-Ferrand, France; 2 Centre de Recherche en Nutrition Humaine d’Auvergne, 58 Boulevard Montalembert, 63009 Clermont-Ferrand, France

**Keywords:** Cancer models, Prostate cancer

## Abstract

One of the most important but less understood step of epithelial tumourigenesis occurs when cells acquire the ability to leave their epithelial compartment. This phenomenon, described as basal epithelial cell extrusion (basal extrusion), represents the first step of tumour invasion. However, due to lack of adequate in vivo model, implication of emblematic signalling pathways such as Ras/Mitogen-Activated Protein Kinase (MAPK) and phosphoinositide 3 kinase (PI3K)/protein kinase B (AKT)/mammalian target of rapamycin (mTOR) signalling pathways, is scarcely described in this phenomenon. We have developed a unique model of basal extrusion in the *Drosophila* accessory gland. There, we demonstrate that both Ras/MAPK and PI3K/AKT/mTOR pathways are necessary for basal extrusion. Furthermore, as in prostate cancer, we show that these pathways are co-activated. This occurs through set up of Epidermal Growth Factor Receptor (EGFR) and Insulin Receptor (InR) dependent autocrine loops, a phenomenon that, considering human data, could be relevant for prostate cancer.

## Abstract (web summary)

The molecular mechanisms leading to basal extrusion are unclear. Here, the authors use the *Drosophila* accessory gland to model human prostate acini and show that Ras/MAPK and PI3K/AKT/mTOR pathways are co-activated in two autocrine loops by dEGF and dIGF, inducing basal extrusion and subsequent tumour formation.

## Introduction

Worldwide, a large majority of cancers originates from epithelial tissues such as lung, breast and prostate^1^. Despite reinforced prevention, most of the tumours are detected at late stages, and patient care is centred on invasive adenocarcinomas, resistant forms of these carcinomas and metastatic carcinomas. As late stages of cancer progression have been under intense scrutiny in the last decades, the molecular mechanisms associated to such progression are largely described, showing for example the major role of receptor tyrosine kinase (RTK)-dependent signalling pathways in these mechanisms. Typically, for prostate adenocarcinoma, the second most common cancer in men, both PI3K/AKT/mTOR pathway and Ras/MAPK pathways are associated with tumour progression^2–4^. In the prostate adenocarcinoma, Ras/MAPK and PI3K/AKT/mTOR pathways display activating genetic alterations in more than 40% of primary tumours and in virtually all metastatic prostate tumours^2^, and phosphoproteomic studies confirmed a strong correlation in the activation of these two pathways^5^. Furthermore, pre-clinical mouse models reproducing alteration of either one or the other pathway in the prostate epithelium display tumourigenesis that mimics histopathological features of the human adenocarcinoma^6,7^. Moreover, advanced tumour progression is obtained when combining alterations in both pathways^8–10^. These different data emphasise that in one hand, Ras/MAPK or PI3K/AKT/mTOR pathways can initiate prostate tumour development, and in the other hand that these pathways are implicated in late phases of tumour progression. However, they explain neither their respective or combined role in actual adenocarcinoma formation nor the molecular mechanisms that could couple these two pathways to promote this phenomenon in vivo.

Adenocarcinoma formation occurs when pre-invasive epithelial cells acquire the ability to leave their epithelial compartment. This implicates that these cells are able to extrude from the normal epithelium and to cross the basement membrane which is the limit of the epithelial compartment. These phenomena can be described as basal extrusion and are resulting in early invasion, as opposed to late invasion associated to the metastatic process. Due to the difficulty to precisely visualise basal extrusion in animals, mechanistic associated to this phenomenon has been essentially described in cellular models or in developing tissues such as *Drosophila* imaginal disc of zebrafish embryo^11–13^, even though Hendley et al. showed the role of P120 catenin in basal extrusion in a mouse model of pancreatic neoplasia^14^. To the best of our knowledge, role of Ras/MAPK pathway in basal extrusion has only been described through the use of Ras^V12^ as an oncogenic hit, and role of PI3K/AKT/mTOR pathway has never been assessed.

To determine the role of Ras/MAPK and PI3K/AKT/mTOR pathways in basal extrusion and understand the underlying mechanisms that may coordinate their hyperactivation in prostate cancer, we have developed a new model of in vivo early invasive adenocarcinoma in the drosophila prostate-like accessory gland. *Drosophila* is a powerful genetic model where more than 70% of genes implicated in human diseases display orthologs^15^ and where Ras/MAPK and PI3K/AKT/mTOR signalling pathways are well conserved^16,17^. *Drosophila* has already proven its pertinence as cancer model for brain^18,19^, lung^20^, and colon^21^. The *Drosophila* accessory gland is a functional equivalent for the prostate, playing a role in fertility by secreting seminal fluid^22,23^. Secretions come from a monolayer of epithelial cells that are well differentiated and quiescent at the adult age, and there is no evidence of stem cells in this tissue. Considering that a majority of prostate adenocarcinoma is thought to originate from luminal cells^24^, epithelial cells from the accessory gland represent a valuable model to study the mechanisms of epithelial prostate tumourigenesis^25,26^.

In this study, we describe this unique model of basal extrusion and tumour formation in the accessory gland that recapitulates most aspects of cancer development. We demonstrate that both Ras/MAPK and PI3K/Akt/TOR pathways are overactivated in the produced tumours, and that these pathways cooperate to induce basal extrusion and subsequent tumour formation. Furthermore, we describe the mechanism that allows the coactivation of these pathways, which relies on the sequential recruitment of a double autocrine feedback loop dependent on Epidermal (EGF/Spitz) and Insulin-like (IGF/Ilp6) Growth Factors and their respective receptors. Finally, using publicly available data of prostate cancer samples^2^ and migration assay in human pre-tumoural prostate epithelial cell line, we assess the possible role of these findings in the actual human pathology.

## Results

### Accessory gland recapitulates prostate microenvironment

**Fig. 1:**
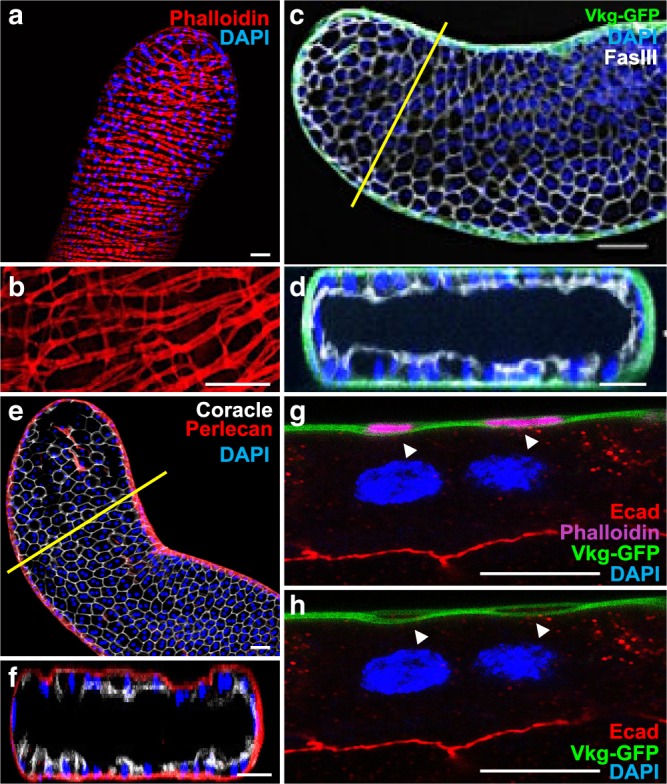
*Drosophila* accessory gland recapitulates prostate epithelial micro- environment. **a**, **b** alexa568-phalloidin (Phalloidin) staining reveals actin accumulation in the well-organised muscle sheet around the gland epithelium. **b** Higher magnification reveals actin bridges between the muscle fibres. This muscle sheet defines a stromal compartment in the gland. **c**–**f** Viking-GFP (GFP tagged Collagen IV, Vkg-GFP in (**c**, **d**) or Perlecan-RFP (Perlecan in (**e**, **f**)) expression reveals the presence of a basement membrane, and Fasciclin III (**c**, **d**) or Coracle (**e**, **f**) staining reveals the apico-lateral domain of the epithelial cells. Transversal optical section (along the yellow line in (**c**) and (**e**)) confirms deposition of Viking-GFP and Perlecan-RFP at the basal pole of the epithelial compartment. **g**, **h** Transversal optical section of the gland at high magnification. Viking-GFP expression reveals the presence of the basement membrane, E-Cadherin (Ecad) staining reveals the apico-lateral domain of one epithelial cell and alexa633- phalloidin (Phalloidin) staining reveals actin accumulation in the muscle fibres. Arrows indicate where the basement membrane encloses the muscle fibres. DAPI (blue) reveals nuclei in (**a**) and (**b**)–(**h**), mostly from binucleated epithelial cells (see **g**–**h**). Representative images in (**a**–**d**, **g**, **h**) from three or more experiments and in (**e**–**f**) from two. Scale bars: 50 μm in (**a**–**f**) and 10 μm in (**g**, **h**).

To realise our study, we first determined whether accessory gland represents a good model to study basal epithelial cell extrusion. Prostate compartment is mostly composed of acini of epithelial cells adjacent to a basement membrane and surrounded by a fibromuscular stroma. It is already known that a layer of muscle fibres completely encloses the accessory gland. Phalloïdin staining reveals this well-organised muscle sheet that forms a continuous layer at the surface of the gland (Fig. 1a). Using higher magnification, we further show that contiguous muscle fibres are linked together by actin bridges, a phenomenon that could explain how mostly unchanged distance between the muscle fibres can be conserved in spite of natural contraction of the fibres (Fig. 1b). Furthermore, we show the presence of a basement membrane along the epithelium, where collagen IV (Viking) and Perlecan can be detected (Fig. 1c–f). This basement membrane encloses the muscle layer (arrow in Fig. 1g, h) which must reinforce the stability of the accessory gland. We conclude that indeed, accessory gland recapitulates classical epithelial microenvironnement as its epithelial cells are adjacent to a basement membrane and closely linked to a stromal-like compartment of muscle cells.

### Oncogene induces epithelial tumourigenesis

**Fig. 2:**
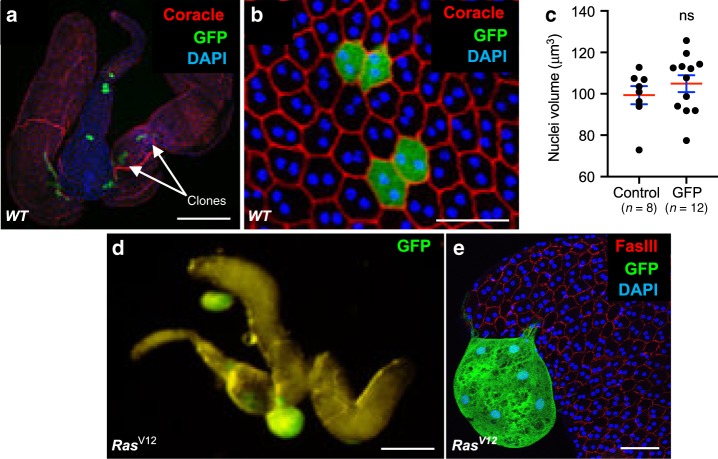
*Drosophila* accessory gland is subjected to oncogene-dependent epithelial cells division and extrusion. (**a**, **b**) and (**d**, **e**) Green fluorescence reveals clones of GFP expressing cells (*WT*). **a**–**c** Clonal cells expressing GFP/nlsGFP only. **d**–**e** Clonal cells co-expressing GFP and Ras^V12^ (*Ras*^*V12*^). a, b Clonal induction is revealed by multifocal localisation of GFP expressing cells (white arrows). Due to time of induction, clones in accessory gland epithelium are composed of two neighbouring cells. These cells do not display phenotype of hypertrophy, as quantified in (**c**) (Mann–Witney test; *P* = 0,3431). Data are represented as mean values ± SEM (*n* = 8 and *n* = 12 accessory glands Control and GFP analysed respectively, from two experiments). **d**, **e** GFP-Ras^V12^ expressing clones are composed of numerous cells and are present outside the gland. DAPI (blue) reveals nuclei in (**a**, **b**) and (**e**). Representative images in (**a**–**e**) from three or more experiments. Scale bars: 200 μm in (**a**) and (**d**), 50 μm in (**b**, **e**).

We then set up experimental conditions to induce epithelial tumourigenesis. Tumourigenesis initiation is thought to start by alteration of one single gene in one single cell. Thanks to genetic tools available in *Drosophila*, we were able to precisely mimic this step by clonal expression of known oncogene Ras^V12^ in a controlled number of cells of the epithelium. Experiments were designed to induce an average of ten clones per gland. Due to time of clonal induction, before last cell division of accessory gland epithelial cells, expression of GFP only (control, Fig. 2a) typically produces clones of two cells that are of same shape and size than non-expressing neighbouring cells (Fig. 2b, c). In contrast, co-expression of GFP and Ras^V12^ was repeatedly correlated to the presence of green masses composed of numerous cells, which clearly appear outside the actual glands. This revealed that Ras^V12^ expression induced clonal epithelial cell hyperproliferation and suggested that some of the clones were able to develop into tumours (Fig. 2d, e).

**Fig. 3:**
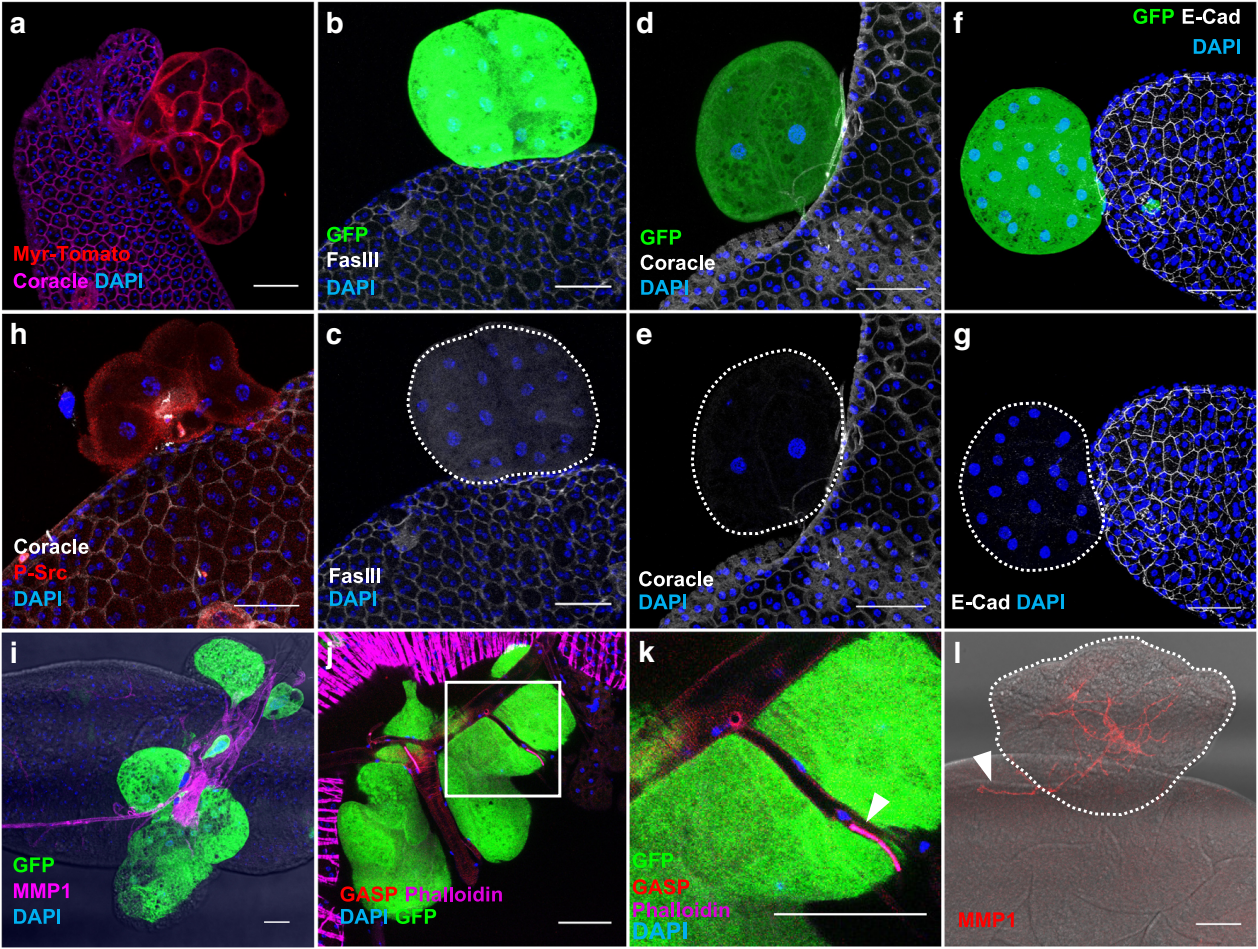
Oncogene expression induces epithelial tumourigenesis in the accessory gland. **a**–**l** GFP-Ras^V12^ expressing clones display hallmarks of cancer. **a** Visualisation of the tumour cell membranes with a myristoylated form of Tomato (Myr-Tomato) indicates that the cells conserve their typical binucleation. **b**–**g** Staining reveals that tumour-like GFP-Ras^V12^ expressing clones lost expression of the epithelial markers. Epithelial markers visualised by staining are: (**b**) Fasciclin III (FasIII), (**d**) Coracle, (**f**) E-Cadherin (E-Cad). **h** Phosphorylated Src (P-Src) is detected at in GFP-Ras^V12^ expressing clones, a feature of mesenchymal cells and/or transformed epithelial cells. **i**–**l** Tracheal system is revealed by Matrix Metalloprotease 1 (MMP1, **i**, **l**) or Gasp (**j**, **k**) staining. **i** Presence of tumour-like GFP-Ras^V12^ expressing clones is correlated to strong hypertrophy of tracheal system. **j**–**k** Gasp staining reveals trachea network associated with tumour-like GFP- Ras^V12^ expressing clones. **k** Zoom of the boxed section in (**j**); white arrowhead points at a strong gasp expression in a low diameter trachea, a feature of developing trachea. **l** MMP1 staining reveals an arborescence of trachea into the tumour and from accessory gland tissue (white arrowhead). DAPI (blue) reveals nuclei in (**a**–**k**). Representative images in (**b**–**i**, **l**) from three or more experiments, in (**j**)–(**k**) from two experiments and in (**a**) from one. Scale bars: 50 μm.

Then, we verified that these masses are actual tumours of epithelial origin. Importantly, these masses are composed of binucleated cells, a specific feature that confirms that they originate from accessory gland epithelial compartment (Fig. 3a). However, these cells are mostly devoid of cell membrane staining for all the epithelial markers we tested: Fasciclin III, Coracle and E-cadherin (Fig. 3b–g). As Fasciclin III and E-Cadherin are adhesion molecules, their absence suggests that clonal cells lost physical contact with surrounding epithelial cells, a phenomenon associated with neoplastic growth in human^27^ and tumour growth in *Drosophila*^28^. Loss of epithelial identity also indicates that Ras^V12^ expressing cells are able to initiate epithelium to mesenchyme transition (EMT), which is considered as a key tumourigenic event in the prostate^29^. Src, a non-receptor kinase, is associated with EMT and cell invasion in cancer^30^, and notably in metastatic progression of prostate adenocarcinoma^31^. Its role in tumourigenesis is assessed as well in *Drosophila* epithelium as in mouse prostate epithelium^32,33^. Phosphorylated Src is present at the membrane of Ras^V12^ expressing cells, defining them as tumour cells (Fig. 3h). Src activation is also correlated to neo-angiogenesis in cancer^31^. In *Drosophila*, a tracheal system provides oxygen to tissues, and growth of this system relies on hypoxic activation by *Drosophila* HIF homolog sima^34^. Therefore, oncogenic expression has been associated to traces of neo-tracheogenesis in this model, which have led to the conclusion that neo-tracheogenesis is the counterpart of neo-angiogenesis in cancer^35^. Ras^V12^ clonal expression is correlated to the presence of overgrown ectopic tracheas (Fig. 3i). These tracheas can be characterised by the use of two different antibodies directed against Gasp and Matrix Metalloprotease 1 (MMP1) (Fig. 3i–l). Gasp, which is implicated in the opening of tracheas during their growth^36^, is strongly expressed in low diameter tracheas at the surface of tumours (arrow in Fig. 3k), but at weaker level in the larger tracheas (Fig. 3j). This shows that these ectopic tracheas are almost entirely mature, i.e. functional, but also still growing, a phenomenon that does not normally occur in adult flies. MMP1 is detected at high levels in the tracheas (Fig. 3i, l), as in embryo and larva in which it is involved in tracheal development^37,38^. This MMP1 staining furthermore reveals that an arborescence of tracheas grows inside the tumours from the tracheas that are normally situated at the surface of the accessory glands (arrow in Fig. 3l). This shows that not only tracheas are growing actively, but also in tight coordination with tumours. We conclude that Ras^V12^ clonal expression induces neo-tracheogenesis in adult accessory gland.

**Fig. 4:**
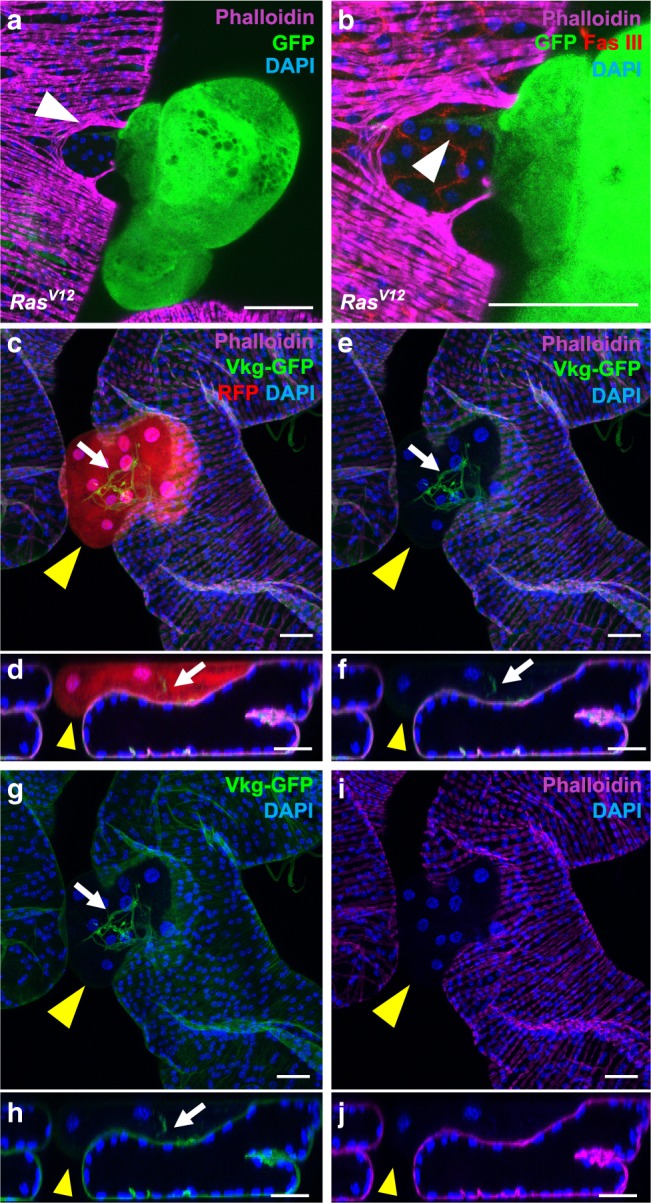
Oncogene expression induces basal epithelial cell extrusion in the accessory gland. **a**, **b** GFP-Ras^V12^ and (**c**–**f**) RFP-Ras^V12^ expressing clones are able to migrate from the epithelial compartment. **a** Alexa633-phalloidin (Phalloidin) staining reveals disorganisation of the muscle fibres at the site of extrusion of GFP-Ras^V12^ expressing clones (white arrow). **b** Zoomed image of (**a**) reveals that the tumour is still anchored to the epithelium (white arrow). **c**, **j** Alexa633-phalloidin (Phalloidin) staining reveals the muscle fibres (**c**–**f**, **i**, **j**) as Viking-GFP staining reveals basement membranes (**c**–**h**). (**d**, **f**, **h**, **j**): optical cross-sections of top panels (respectively, **c**, **e**, **g**, **i**). Staining reveals that RFP-Ras^V12^ tumour develops outside the epithelial compartment, as it is not enclosed by a basement membrane or a muscle layer (yellow arrows). Basement membrane staining reveals tracheal branches inside the tumour (white arrows). DAPI (blue) reveals nuclei in (**a**–**j**). Representative images from two experiments. Scale bars: 50 μm.

Then, we wanted to definitively confirm that we are in the presence of a phenomenon of basal extrusion. First, we noticed that the presence of tumours is linked to defects in the muscle layer, reflecting the probable migration of the clonal epithelial cells through this muscle sheet (arrow in Fig. 4a), especially as tumours frequently stay attached to the normal epithelium (arrow in Fig. 4b). Then, we revealed in the same experiments tumours in red (RFP), basement membrane (viking-GFP in green^39^) and the stromal compartment of muscle cells (phalloïdin staining in magenta) (Fig. 4c–j). Tumours appear outside both the epithelial and the stromal-like compartment of muscle cells (yellow arrows in c–f, i, j). Surface of the tumours display no evident basement membrane (yellow arrows in Fig. 4c–h) and only basement membrane within these masses corresponds to tracheas that grew inside the tumours (white arrows in Fig. 4c–h).

Together, our results demonstrate that expression of a known oncogene in clones of accessory gland epithelial cells induces epithelial cell extrusion and early invasion resulting in the formation of adenocarcinoma-like tumours. Due to the shape and structure of accessory gland, it is also particularly easy to visualise the tumours directly at the dissection, allowing easy quantification of this basal extrusion phenomenon.

### Invasive tumours display RTK-dependent pathways coactivation

**Fig. 5:**
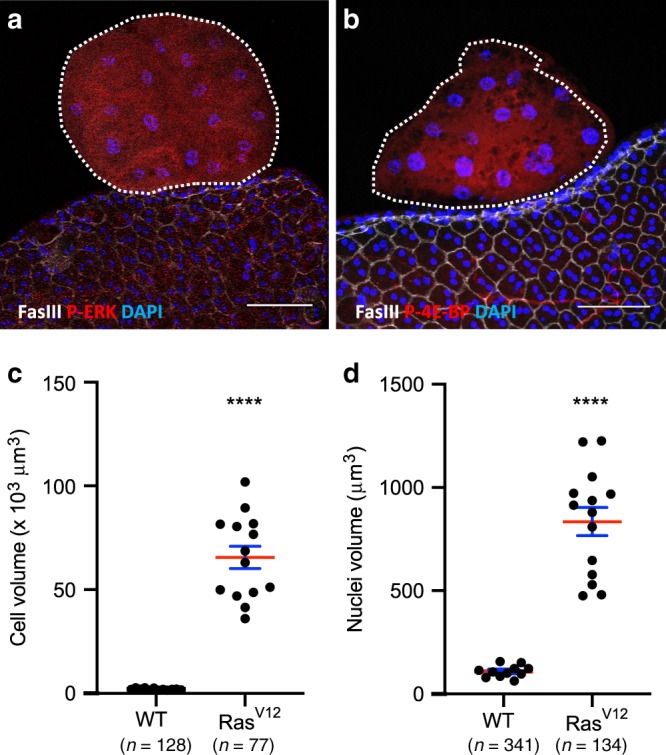
Ras/MAPK and PI3K/AKT/TOR pathways are concomitantly activated in Ras^V12^ expressing tumours. GFP-Ras^V12^ expressing tumours are encircled by dotted lines. Fasciclin III (FasIII) staining reveals normal epithelial cells. **a** GFP-Ras^V12^ expressing tumours display phosphorylation of ERK, a downstream target of the Ras/MAPK pathway. **b** GFP-Ras^V12^ expressing tumours display phosphorylation of 4E-BP, a downstream target of the PI3//AKT/mTOR pathway. **c**, **d** Comparison of cell (**c**) and nuclei (**d**) volumes of tumour cells with wild-type cells (WT) (**** Unpaired *t* test; *P* < 0.0001) reveals strong hypertrophy of GFP-Ras^V12^ expressing cells (Ras^V12^). Data are represented as mean values±SEM (cell volume: *n* = 12 and *n* = 14 accessory glands Control and Ras^v12^ analysed, respectively, from seven experiments: nuclei volume: *n* = 11 and *n* = 14 accessory glands Control and Ras^v12^ analysed respectively, from seven experiments). Representative images in (**a**, **b**) from three or more experiments. DAPI (blue) reveals nuclei in (**a**, **b**). Scale bars: 50 μm.

Our aim was then to determine if accessory glands tumours were able to reproduce in *Drosophila* this specific feature of prostate adenocarcinoma which is the co-deregulation of Ras/MAPK and PI3K/AKT/mTOR pathways^2,5^. To test the activity of the two considered pathways, the phosphorylation of two downstream targets, ERK (Rolled) and 4E-BP, was assessed. In response to Ras^V12^ expression, P-ERK is logically detected in tumours (Fig. 5a) which indicates canonical Ras/MAPK pathway activation. In the tumours, cells display up to 40 times their normal size (see Fig. 3a for membrane staining and Fig. 5c for quantification), showing that their growth is abnormally activated. Correlatively, they display oversized nuclei (Fig. 5d), indicating a probable phenomenon of endoreplication. In *Drosophila*, cell size is tightly controlled by TOR activity^17,40^. Indeed, 4E-BP phosphorylation is detected in tumour cells (Fig. 5b), indicating a co-recruitment of the PI3K/AKT/TOR pathway in these cells.

### RTK-dependent pathways coactivation drives basal extrusion

**Fig. 6:**
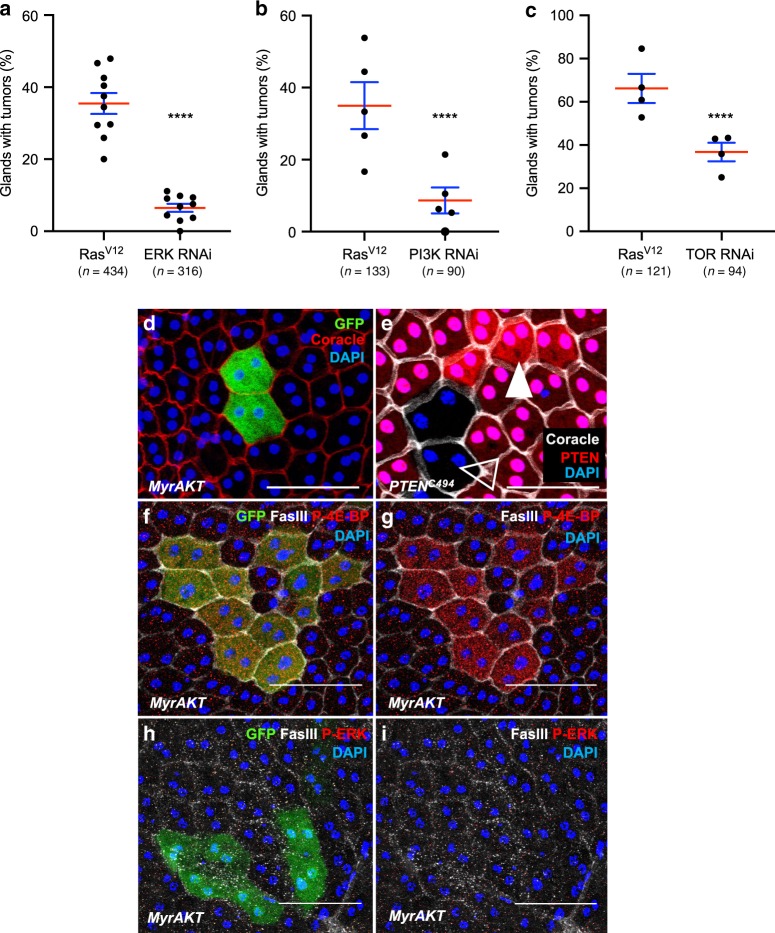
Ras/MAPK and PI3K/AKT/TOR pathways are both necessary for tumour formation. **a**–**c** Invasive tumour frequency of GFP-Ras^V12^ expressing clones (Ras^V12^) is impaired by downregulation of ERK (ERK RNAi in (**a**)), of PI3K *(*PI3K RNAi in (**b**)) and of TOR (TOR RNAi in (**c**)) (Chi^2^ test; ****i < 0.0001 in (**a**) and (**b**), ***P = 0.0023 in (**c**)). Data are represented as mean values ± SEM. (**d**) Overactivation of PI3K/AKT/TOR pathway in GFP-myristoylated-Akt (MyrAkt) expressing clones induces mild hypertrophy compared to surrounding cells but no tumour formation. (**e**) Generation of *PTEN*+*/*+ cells (white arrow, high expression of nlsRFP) and *PTEN−/−* cells (empty arrow, no nlsRFP) in *PTEN*+*/−* accessory gland reveals a correlation between *PTEN* copy number and cell size. **f**–**i** GFP-MyrAkt expressing clones display phosphorylation of 4E-BP (**f**, **g**) but not of ERK (**h**, **i**), revealing a lack of recruitment of the Ras/MAPK pathway in these cells. DAPI (blue) reveals nuclei in (**d**–**i**). Representative images in (**d**–**g**) from three or more experiments, in (**h**, **i**) from two. Scale bars: 50 μm.

We then wanted to determine whether the activated pathways were implicated in the process of invasion itself. So, we quantified invasive tumour frequency for different combinations of activation/inactivation of the two considered pathways. Expression of ERK RNAi in the Ras^V12^ expressing cells significantly decreases invasive tumour frequency (i.e. clones that left the epithelium compartment to form external masses) (Fig. 6a), showing that Ras^V12^ oncogenic activity depends on the canonical Ras/MAPK pathway. Similarly, expression of PI3K RNAi or TOR RNAi in the Ras^V12^ expressing cells significantly decreases invasive tumour frequency (Fig. 6b, c), showing that Ras^V12^ oncogenic activity depends also on the PI3K/AKT/TOR pathway in the accessory gland.

PI3K/AKT/TOR pathway can be directly activated either by *phosphatase and tensin homolog* (*pten)* deletion or by expression of a myristoylated form of Akt (myr-Akt). The use of one or the other of these genetic modifications does not induce tumour development, but only mild cell hypertrophy is observed (Fig. 6d, e). Furthermore, myr-Akt expressing clones display 4E-BP phosphorylation (Fig. 6f, g), but no ERK phosphorylation (Fig. 6h, i), showing that PI3K/AKT/TOR overactivation does not induce Ras/MAPK pathway. Thus, it indicates that a failure to co-recruit the two pathways correlates with a lack of basal extrusion in the accessory gland.

From our data, we conclude that both the canonical Ras/MAPK pathway and the PI3K/AKT/TOR pathway are necessary for cells to evade the epithelial compartment, and that lack of recruitment of one or the other pathway impairs the basal extrusion and subsequent tumour formation.

### Tumour invasion depends on an EGF/EFGR autocrine loop

**Fig. 7:**
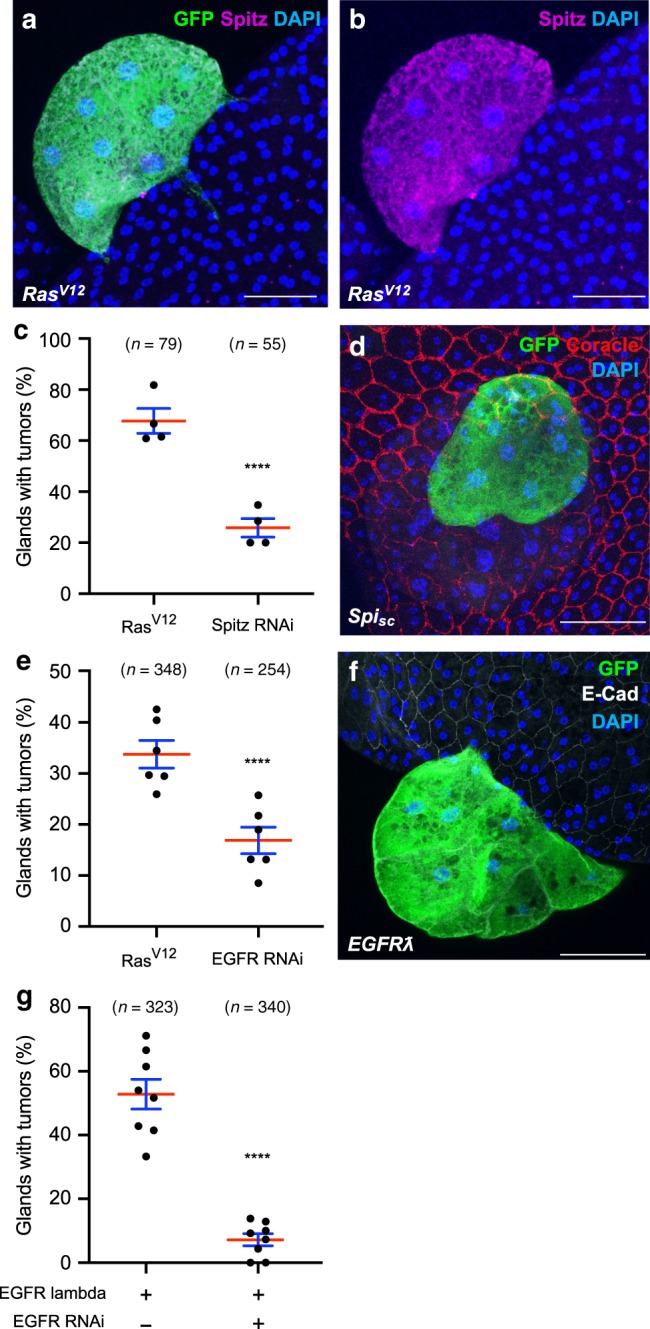
Ras^V12^-driven tumour formation depends on the setting of an EGF/EFGR autocrine activation loop. **a**, **b** Immunostaining reveals that EGF/Spitz (Spitz) is expressed in GFP-Ras^V12^ expressing clones. **c** Invasive tumour frequency of GFP-Ras^V12^ expressing clones (Ras^V12^) is impaired by downregulation of *EGF/Spitz* (Spitz RNAi) (Chi^2^ test; *P* < 0.0001). **d** Clonal expression of a secreted form of EGF/Spitz (Spi_sc_) leads to tumour formation. **e** Invasive tumour frequency of GFP-Ras^V12^ expressing clones (Ras^V12^) is impaired by downregulation of EGFR (EGFR RNAi) (Chi^2^ test; *P* < 0.0001). **f** Clonal expression of a constitutively active form of EGFR (EGFRƛ) induces the formation of tumours. **g** Invasive tumour frequency of GFP- EGFRƛ expressing clones (EGFRƛ) is strongly impaired by downregulation of Ras (Ras RNAi) (Chi^2^ test; *P* < 0.0001). Data are represented as mean values±SEM. Representative images in (**a**, **b**, **d**, **f**) from three or more experiments. ****P < 0.0001. Scale bars: 50 μm.

**Fig. 8:**
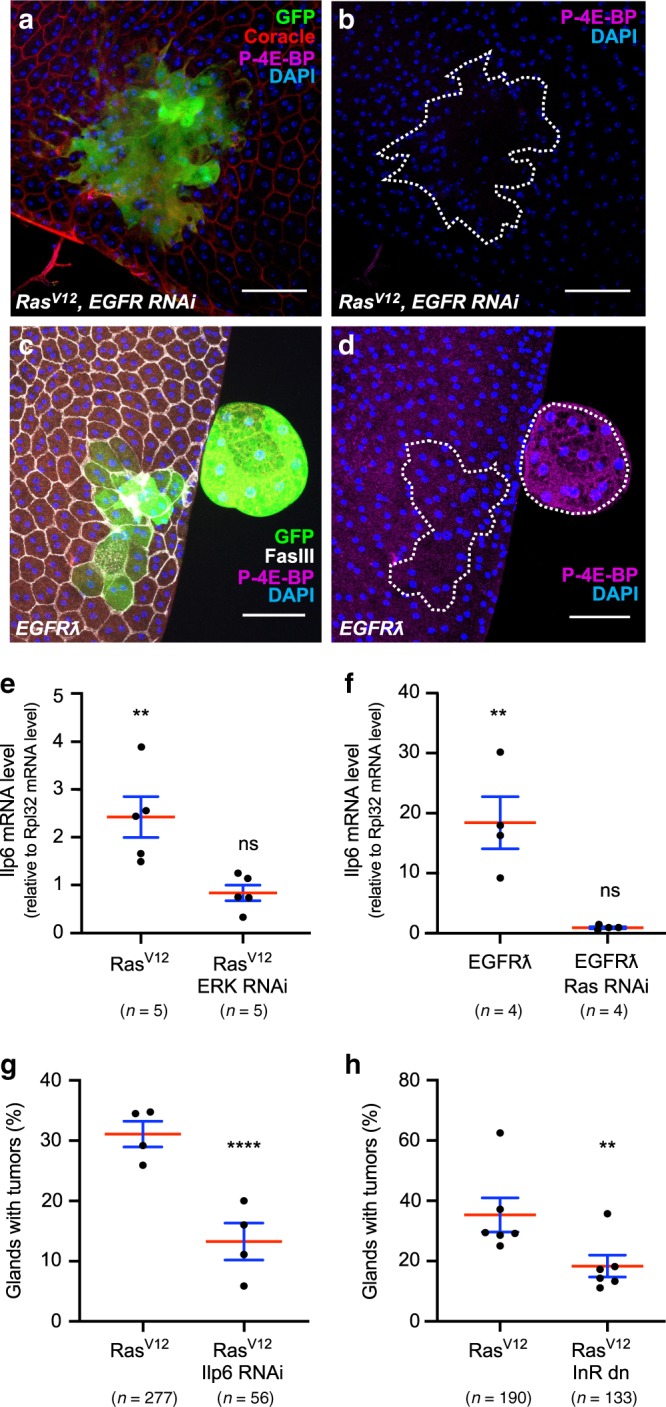
Ras^V12^-driven tumourigenesis depends on the setting of an IGF/InR autocrine activation loop. **a**, **b** Phosphorylation of 4E-BP, a downstream target of the PI3//AKT/mTOR pathway, is not detected in Ras^V12^, EGFR RNAi expressing clones. **c**, **d** Phosphorylation of 4E-BP is detected in tumours induced by clonal expression of a constitutively active form of EGFR (EGFRl). **e** qPCR quantification reveals that *IGF/Ilp6* (*Ilp6*) is overexpressed in glands displaying GFP-Ras^V12^ expressing clones compared to control gland (left column, Two tailed Mann–Whitney test: *P* = 0,0079. RasV12-dependent overexpression of IGF/Ilp6 is suppressed by downregulation of ERK (ERK RNAi, right column, Two tailed Mann–Whitney test: *P* = 0,68). **(f)** qPCR quantification reveals that *IGF/Ilp6* (*Ilp6*) is overexpressed in glands displaying GFP-EGFRl expressing clones compared to control gland (left column, Two tailed Mann–Whitney test: *P* = 0,0079. EGFRl-dependent overexpression of *IGF/Ilp6* is suppressed by downregulation of Ras (Ras RNAi, right column, Two tailed Mann–Whitney test: *P* = 0,21). **g** Invasive tumour frequency of GFP-Ras^V12^ expressing clones (Ras^V12^) is impaired by downregulation of IGF/Ilp6 (Ilp6 RNAi) (Chi^2^ test; *P* < 0.0001). **h** Invasive tumour frequency of GFP-Ras^V12^ expressing clones (Ras^V12^) is impaired by co-expression of a dominant-negative form of InR (InR dn) (Chi^2^ test; *P* < 0.0001). Data are represented as mean values ± SEM. Representative images in (**a**–**d**) from three or more experiments. **P < 0.01; ****P < 0.0001. Scale bars: 50 μm.

Then, we decided to determine how the pathways are recruited during tumourigenesis in the accessory gland. Considering that, in prostate cancer, a majority of primary tumours display no activating mutation in the considered pathways^2^, we searched for an alternative mechanism. Classical activation of the PI3K/AKT/mTOR and the Ras/MAPK pathways is thought to mostly rely on growth factors. Interestingly, Ras^V12^ clones overexpress EGF/Spitz, an EGF/TGFα homolog and ligand of *Drosophila* EGFR^41^ (Fig. 7a, b). EGFR is upstream of both Ras/MAPK and PI3K/AKT/TOR pathways in mammals^42^, but could be more specific to Ras/MAPK pathway in *Drosophila*^43^. Strikingly, suppression of EGF/Spitz by RNAi expression in Ras^V12^ clones strongly reduces invasive tumour frequency (Fig. 7c), showing that expression of this ligand sustains Ras^V12^-driven basal extrusion in the accessory gland. Moreover, if clonal overexpression of a secreted form of EGF/Spitz (Spitz_sc_)^44^ in epithelial cells mostly induces a high rate of mortality during pupal stage, the remaining adult flies can display tumours exhibiting the same hallmarks of cell overgrowth and loss of epithelial markers as in Ras^V12^ induced tumours (Fig. 7d). These results agree with the existence of an EGFR-dependent autocrine feedback loop that is necessary for Ras^V12^ oncogenic transformation. Logically, co-expression of an EGFR RNAi in Ras^V12^ clones strongly decreases invasive properties of developed tumour (Fig. 7e), confirming the role of EGFR in Ras^V12^-driven tumourigenesis. Furthermore, expression of a constitutively active form of EGFR, EGFRλ, leads to the formation of tumours that display the same hallmarks as in Ras^V12^ induced tumours (Fig. 7f), showing that overactivated EGFR can induce basal extrusion of epithelial cell in the accessory gland. Finally, co-expression of a Ras RNAi in EGFRλ clones strongly decreases their invasive properties (Fig. 7g), confirming the role of Ras/MAPK signalling pathway in the EGFR-dependent tumourigenic process. Interestingly, observation of epithelial clones co-expressing EGFR RNAi and Ras^V12^ reveals that they can be composed of many cells (observe the extension of the clone in Fig. 8a), suggesting that EGFR recruitment is less required for cell division than for cell extrusion.

We conclude that upon Ras^V12^ dependent initial activation, Ras/MAPK pathway must be re-activated by an EGF/EGFR autocrine loop to induce cell invasive capacities, definitively showing that Ras/MAPK pathway is necessary for basal extrusion independently of the mechanism of oncogenic initiation.

### Tumour invasion depends on an IGF/InR autocrine loop

We then wanted to determine how PI3K/AKT/TOR pathway is recruited. At first, we observed that P-4E-BP staining is never detected in Ras^V12^/EGFR RNAi clones, and cells stay smaller in this condition compared to Ras^V12^ expressing cells (Fig. 8a, b), indicating a lack of recruitment of the PI3K/AKT/TOR pathway in these clones and suggesting that EGFR could be implicated in the recruitment of this pathway. However, we also observed that clonal expression of EGFRλ induces PI3K/AKT/TOR pathway recruitment, as for Ras^V12^ expression, but this recruitment and associated cellular hypertrophy occurs only in external cell masses (Fig. 8c, d). This suggests that PI3K/AKT/TOR pathway is not recruited directly by EGFR, but via a molecular intermediate. In *Drosophila*, PI3K/AKT/TOR pathway is mainly recruited by the Insulin Receptor (InR)^45^, and seven ligands are known to activate InR. We then searched for their expression. Both Ras^V12^ and EGFRλ clonal expression in the accessory gland induces mRNA overexpression of one ligand, IGF/Ilp6 (left columns in Fig. 8e, f). Indeed, we decided to determine the potential role of IGF/Ilp6 overexpression in Ras^V12^-driven tumourigenesis. Strikingly, downregulation of IGF/Ilp6 (Ilp6 RNAi) in Ras^V12^ clones significantly decreases invasive tumour frequency (Fig. 8g). Logically, co-expression of a dominant-negative form of InR (InR dn) in Ras^V12^ clones also decreases invasive tumour frequency (Fig. 8h), showing that this receptor is also implicated in Ras^V12^ oncogenic activity, and then defining a second autocrine loop that participates to accessory gland invasive tumour formation.

Next, we decided to precise which molecular actors are necessary for this *IGF/Ilp6* overexpression. First, we co-expressed Ras RNAi with EGFRλ. It completely abolishes IGF/Ilp6 mRNA overexpression compared to EGFRλ expression alone, showing that this overexpression depends on Ras activation (right column in Fig. 8f). Second, we co-expressed ERK RNAi with Ras^V12^. This also completely abolishes IGF/Ilp6 mRNA overexpression compared to Ras^V12^ expression alone, demonstrating that *IGF/Ilp6* overexpression depends also on ERK activation (right column in Fig. 8e). We conclude that IGF/Ilp6 mRNA overexpression exclusively depends on the activation of the classical EGFR/Ras/MAPK pathway.

### *EGF* is specifically overexpressed in primary prostate cancer

**Fig. 9:**
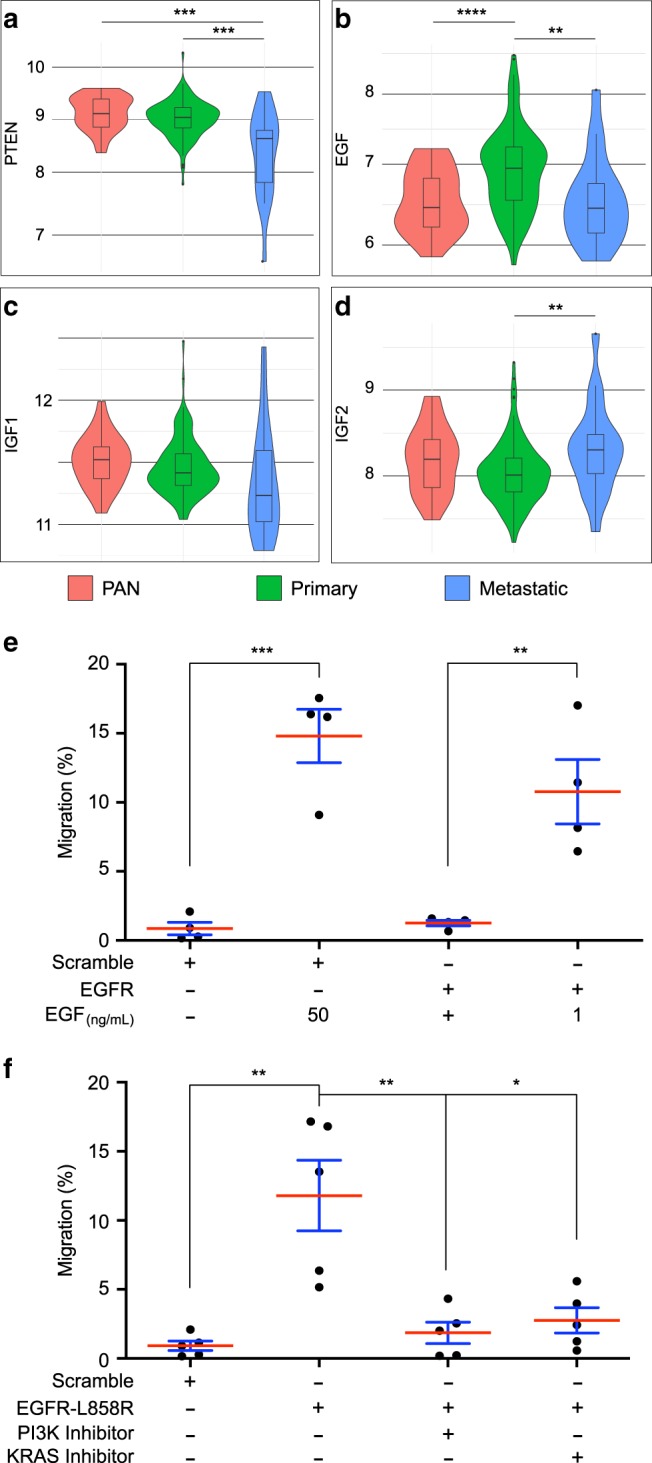
*EGF* expression is specifically increased in primary prostate cancer. **a**–**d** Violin plots showing mRNA expression data for five genes in normal prostate tissues (PAN, red), primary prostate tumours (PRIMARY, green) and metastatic prostate tumours (Metastatic, blue). Expression data were first published by Taylor et al.^2^. Unpaired *t* test, (**a**), *P* < 0.001 (PAN vs metastasis), *P* = 0.0022 (primary vs metastasis); (**b**) *P* < 0.001 (PAN vs primary), *P* = 0.0089 (primary vs metastasis); (**d**) *P* = 0.0364 (primary vs metastasis). **e** Migration of P69 cells is promoted by EGF dependently of EGFR. Data are represented as mean values* ±*SD, unpaired *t* test, *n* = 4 independent experiments. **P = 0.0066; ***P = 0.0004. **f** Migration of P69 cells is induced by expression of constitutive form of EGFR (EGFR-L858R) and this migration is impaired by specific inhibition of either PI3K or KRAS activity. Data are represented as mean values* ±*SD, unpaired *t* test, *n* = 5 independent experiments. *P = 0.0104; **P = 0.0058. *P < 0.05; **P < 0.01; ***P < 0.001; ****P < 0.0001.

Finally, to determine whether our findings could be relevant to prostate cancer, we decided to examine *EGF* and *IGFs* expression in human samples. We selected data where mRNA levels were available in normal, primary and metastatic samples^2^. First, we determined whether these mRNA could reflect alterations that are hallmarks of tumour progression. Typically, advanced prostate cancers are associated to loss of *PTEN* expression^46^. In the used cohort, PTEN mRNA expression tends to decrease in primary tumours when compared to normal tissue (*p* = 0.07) and significantly decreases in metastatic samples compared to normal tissue (*p* = 0.00047) and primary tumours (*p* = 0.002) (Fig. 9a). Thus, *PTEN* expression is, as expected, inversely correlated with tumour grade. We then considered the expression of *EGF*, *IGF1* and *IGF2* (Fig. 9b–d). EGF mRNA expression presents a bell-shaped pattern (Fig. 9b): it is significantly increased in primary tumours compared to both normal (*p* = 2 × 10^−5^) and metastatic (*p* = 0.0089) tissues which are expressing very similar levels of *EGF* (*p* = 0.92). Furthermore, this expression increases even in primary tumours with the lowest available Gleason score (Gleason score = 6, *p* = 0.0012). In contrast, *IGF1* expression does not significantly change between the considered conditions (Fig. 9c), and *IGF2* expression tends to decrease in primary tumours compared to normal tissue (*p* = 0.05) and significantly increases in metastatic samples compared to primary tumours (*p* = 0.0036) (Fig. 9d). Together, these results indicate that EGF mRNA is specifically overexpressed during early carcinogenesis, suggesting that EGF could play a role in this particular stage of cancer.

Then, we decided to check the role of the EGFR signalling pathway in P69, a cell line derived from immortalisation of human normal prostate epithelial cells^47^. Using transwell chamber assay, we show that supraphysiological doses of EGF, or lower doses in a context of EGFR overexpression, significantly increase P69 cells migration (Fig. 9e). Furthermore, expression of a constitutively active form of EGFR (EGFR-L858R^48^) induces similar migration of P69 cells in the absence of EGF, and this migration is impaired by treatment with specific inhibitors of either KRAS^49^ or PI3K (Fig. 9f). We conclude that, as demonstrated in *Drosophila*, migration of human pre-tumoural prostate epithelial cells depends on EGFR signalling and downstream activation of both Ras/MAPK and PI3K/Akt/mTOR pathways.

**Fig. 10:**
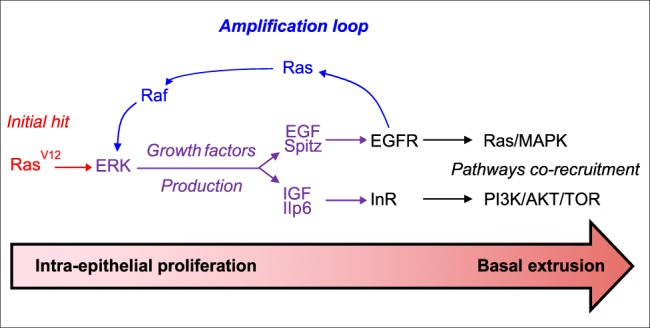
Ras/MAPK and PI3K/AKT co-recruitment is necessary for tumour extrusion. Initiation by a oncogenic hit (red) allows ERK activation in rare epithelial cells. This induces production of two growth factors in these cells, EGF/Spitz and IGF-Ilp6 (violet). EGF/Spitz re-activates ERK in an autocrine manner, via EGFR activation (amplification loop, blue). This sustains an increase in the growth factors production. Subsequently, EGFR/InR autocrine activation induces a coordinated recruitment of the Ras/MAPK and PI3K/AKT/TOR pathways. This finally allows formation of invasive tumours outside the epithelial compartment.

So, collectively, our data show that accessory gland tumourigenesis, and more precisely basal extrusion, relies on a double autocrine loop: the first one depends on EGF/Spitz and its receptor EGFR to amplify Ras/MAPK pathway activation; the second one depends on Ras/MAPK-dependent production of IGF/Ilp6 to recruit the PI3K/AKT/TOR pathway via InR activation. And this coactivation is necessary in a cell-autonomous manner for the epithelial cells to be able to leave the epithelial compartment to form adenocarcinoma-like tumours (Fig. 10).

## Discussion

More than 95% of prostate cancers are of epithelial origin and are thought to mainly arise from a single cell^24^. To faithfully reproduce what is thought to happen in the earliest stages of tumour formation in patients, we have decided to produce a single genetic alteration in few clones of randomly selected and mostly differentiated cells. Furthermore, we show that accessory gland epithelium is adjacent to a basement membrane, is surrounded by a stromal-like sheet of muscle fibres, and that oncogene-induced epithelial cells are able to cross both layers to form external tumours (Figs. 1 and 4). This recapitulates the phenomenon of basal epithelial cell extrusion, which is thought to be central to cell invasion^11,50^. Basal extrusion has been described in cell culture^50^, in *Drosophila* imaginal disc^51^, in zebrafish embryo^13^ and once in mouse^14^. However, to the best of our knwowledge, implication of Ras/MAPK and PI3K/AKT/mTOR pathways has never been assessed in this phenomenon, despite the fact that these pathways are among the most deregulated in cancers^52,53^, and especially in epithelial cancers^54–57^ such as prostate adenocarcinoma^58^. Here, we show in a new model of accessory gland tumourigenesis that both pathways are implicated in basal extrusion, indicating that this step demands a particular state of activation for the cell that undergoes this basal extrusion. Furthermore, this finding correlates with the fact that the two considered pathways are already frequently co-deregulated in primary tumours^2^. From our experiments, where oncogene expression is restricted to few cells and intra-tumoural inhibition of the pathways decreases invasion, we can infer that the mechanisms of basal cell extrusion are cell autonomous, as previously shown in cell lines^50^. Indeed, we show that this cell-autonomous mechanism relies on the production of two growth factors, and subsequent activation of two autocrine loops (Fig. 10). Role of autocrine loops has been hypothetized in late tumourigenesis, as higher levels of growth factors have been found in tumoural tissues^59,60^, and has been studied in cell models where inhibition of these loops decreases tumourigenic features such as migration or proliferation capacity^61,62^ as their activation have been linked to transformation of various epithelial cells (reviewed in ref. ^63^). However, to the best of our knowledge, role of autocrine loops has never been demonstrated for basal extrusion in vivo. If these loops seem implicated in tumour late progression, so could they be more important for early human tumour development. In fact, many strategies have been attempted to treat cancer patients especially by blocking EGF/EGFR autocrine loop. However, for advanced prostate cancer, these strategies have shown poor results, as well for monotherapies^64,65^ as for combined treatments with classical anti-prostate cancer agents^66,67^. It could be logical if autocrine loops are less implicated in late stages of cancer but more in the capacity for tumour cells to leave the epithelial compartment. In later stages, higher rates of activating mutations in the Ras/MAPK and PI3K/AKT/mTOR pathways could suppress the need for RTK-driven activation. In contrast, in early tumourigenesis, as fewer genetic alterations are present, activation of signalling pathways must rely on different mechanisms. As we show it in the accessory gland, this recruitment could be efficiently done in tumour cells by autocrine production of growth factors, autocrine activation of their RTK and subsequent activation of the pathways necessary for the tumour development. In a human cohort of prostate cancer samples, we found that *EGF* is more expressed in primary tumours than either in normal tissue or in metastases. This could correlate with an early requirement for such growth factor in the formation of adenocarcinoma. Contrary to our observations in *Drosophila*, no early overexpression of *IGFs* can be detected in human samples. However, in human, EGFR is able to recruit both Ras/MAPK and PI3K/AKT/mTOR pathway^42^, and *EGF* overexpression could drive their activation and act in the same way as EGF/Spitz and IGF/Ilp6 in *Drosophila*.

To study early phases of tumourigenesis remains difficult in vivo, especially for epithelial cells that can develop into benign tumours still in the epithelial compartment such as benign prostatic intraepithelial neoplasia, or into adenocarcinoma that are characterised by an expansion out of the epithelial compartment^68^. The model we developed in the *Drosophila* accessory gland represents a unique in vivo model to explore basal extrusion and early invasion. We were able to show that two major pathways of cancer progression are implicated in this basal extrusion, and to demonstrate that these two pathways are co-recruited by autocrine loops (Fig. 10). Further investigation will be necessary to test whether other pathways implicated in late tumourigenesis are important in this phenomenon. Furthermore, it will also be important to determine which genes are activated or inhibited by these pathways and which mechanisms are recruited to promote the actual extrusion.

## Methods

### Fly stocks and experimental crosses

*y,w,HS:flp122/+;Act:FRTstopFRTGal4,UAS:GFP/CyO* flies allowed conditional clonal expression of GFP. When combined to *UAS*:*Ras*^*V12*^ flies (4847) or *UAS:EgfrλTop* (59843), they allowed conditional clonal co-expression of *GFP* and *Ras*^*V12*^ (*GFP-Ras*^*V12*^ flies) or *Egfr*λtop (*GFP-Egfrλ* flies). *GFP-Ras*^*V12*^ and *GFP-Egfrλ* flies were then crossed with following stocks to realise experiments: *UAS-GFP.nls* (4775), *UAS-TorRNAi* (35578), *UAS-SpiRNAi* (34645), *UAS-Pi3kRNAi* (61182), *UAS-MyrTomato* (32221), *UAS-RolledRNAi* (34855), *UAS-Egfrλtop* (59843), *UAS-RasRNAi* (106642) *UAS-InRK*^*1409A*^ DN (8252), *UAS-Ilp6RNAi* (33684), *UAS:s-Spi* (58436) from the Bloomington Stock Center and *UAS-EgfrRNAi* (43267) from Vienna Drosophila Resource Center). *y,w,HS:flp122/+;Act:FRTstopFRTGal4,UAS:GFP/CyO;UAS-myr-AKT/TM3Sb* were also used to induce AKT/TOR pathway activation (*GFP-MyrAkt* flies). Other stocks used: *HS:flp122/+;FRT40A,PTEN*^*C494*^*/FRT40A,Ubi:nlsRFP*.

### Conditional expression induction

Briefly, condition of use of the flippase (flp)/FRT system was determined to produce an average of 4–6 clones per accessory gland (≈1% of total number of epithelial cells). Flippase-dependent recombination was induced during pupal stage by 12 min (for GFP-Ras^V12^ and GFP-EGFRλ flies) or 20 min (for GFP-MyrAkt flies) heat-shock at 37 °C. Flies were then kept at 25 °C until the end of pupal stage. Males were collected at emergence from pupae 3 to 3.5 days after heat shock and kept for another 3 days at 25 °C before dissection.

### Immunohistochemistry and imaging

Accessory glands were dissected in 1X PBS or 1X PBS containing phosphatase inhibitors (orthovanadate 1 mM, β-glycerophosphate 20 mM and NaF 1 mM) for the detection of phosphorylated proteins. They were fixed for 10 min in 4% paraformaldehyde, washed once with 1X PBS and three times with 1X PBS containing 0.2% Triton (PBS-T) for permeabilization. The samples were blocked for 10 min with 0.5% of bovine serum albumin in PBS-T and incubated overnight at 4°C in primary antibodies diluted in the same blocking solution. The tissues were then washed twice for 5 min with PBS-T and incubated for 1 h at room temperature in secondary antibody diluted 1:1000 in blocking solution. Added with secondary antibodies, DAPI (Di AminidoPhenylIndol, D8417, Sigma) 1:1000 was used to stain DNA and 1/5000 Alexa568-phalloidin (A12380, Life Technology) or Alexa633-phalloidin (A22284, Life Technology) were used to reveal F-actin. Following two washes in PBS-T, the samples were then mounted in Vectashield (#-1000, Vector Laboratories) and visualised using a Leica SP5 or SP8 confocal microscope. Image stacks were processed in ImageJ or Imaris software.

We used the following antibodies: Mouse Coracle (1:300, #C566.9 DHSB), mouse Fasciclin III (1:400, #7610 DHSB), rat E-Cadherin (1:1000, #DCAD2 DHSB), rabbit P-4E-BP1 (1:200, 2855S Cell Signalling), rabbit P-ERK (1:500, 4370S Cell Signalling), goat GFP (1:1000, #5450 Abcam), mouse GASP (1/5, 2A12 DHSB), mouse MMP1 (1/100, 14A3D2 DHSB; 1:10 #3A6B4 #3B8D12 #5H7B11 DHSB), rat Spitz (1:100, DHSB), rabbit P-Src (1:500, #44-660G Invitrogen), secondary antibodies coupled to different fluorophores 488 (1:1000, A11055 Invitrogen), Cy5 or Cy3 (1:1000, 711-165-152, 711-175-152, 712-165-153, 712-175-153, 715-165-150, 715-165-151, 715-175-150, 715-175-151, Jackson Immunology).

### RNA extraction, cDNA synthesis and Real-time Quantitative PCR

RNAs were isolated from accessory glands dissection, by Trizol-extracted total accessory glands (Invitrogen). Reverse transcription was performed by using SuperScript IV Reverse Transcriptase kit (ThermoFisher Scientific). Quantitative PCR was performed using on the LC480. *rpl32* was used for the normalisation.

### Primers for qRT-PCR analysis were

Dilp6^69^: forward: 5′ TGCTAGTCCTGGCCACCTTGTTCG-3′; reverse: 5′ GGAAATACATCGCCAAGGGCCACC-3′

Rpl32^70^: forward: 5′-TTGGCTTCGGTTTCCGGCAAG-3′; reverse: 5′-ATCGATCCGACTGGTGGCGGAT3′

### Cells and nuclei size

Nuclei and nucleus volume were determined from 3D reconstruction with Imaris software. Cells area was determined with direct measure of contour cells in Image J Software.

### Invasive tumour frequency

During dissection, direct observation under binocular microscope allowed to visualise the presence of tumours at the surface of the accessory glands. Tumour frequency was determined as the percentage of flies that displayed at least one tumour on their accessory glands. Following preliminary tests, it is important to note that we designed the experiments to obtain a similar number of UAS in all the flies we tested to avoid Gal4 titration effect; to avoid a bias in clonal induction, for each repeat of each experiment, all procedures were done in the same time for all the genotypes compared in the experiment (as visualised by identical number of dots for each condition in the different panels).

### Cell culture, transfection and migration assay

Pre-tumoral human prostate epithelial cells (P69) were cultured in RPMI-1640 medium (Invitrogen, USA) with penicillin/streptomycin (100 mg/ml) (Invitrogen, USA), l-glutamine (2 mM) (Invitrogen, USA) and supplemented with 10% of foetal bovine serum (Eurobio, France). All cells were grown at 37 °C in a humified chamber with 5% CO_2_.

Transient plasmid DNA incorporation was performed on P69 cells. pGFP (scramble), pEGFR and pGFP-EGFR-L858R vectors were obtained from Addgene and transfection were performed using jetPEI^®^ transfection reagent (Polyplus transfection, France), according to manufacturer´s protocol. After transfection and serum starvation overnight, cell migration ability was challenged in transwell chambers of 8-mm pores (Corning, USA), followed by addition in the lower chamber of recombinant human-EGF (Gibco, USA) at 1 or 50 ng/ml in serum-free RPM1 medium or in some experiments in the upper chamber with PI3K inhibitor LY294002 (2 µM) (Cell signalling technology) or KRAS inhibitor SAH-SOS1A (10 µM) (EMD Millipore, USA) in serum-free RPM1 medium.

### RNA-seq data

We retrieved processed RNA-seq data from the website http://cbio.mskcc.org/cancergenomics/prostate/data/.

We only considered already treated and normalised log2 expression data.

### Violin Plots

Violin plot were made using R package “ggplot2” applying “geomviolin()” function. “geomboxplot()” function was used to add boxplot inside violin.

### Statistical analyses

All experiments were done a minimum of four times (independent experiments, values represented as dots in the graphs) on numerous glands, or cells for Figs. 2b and 5c, d (total numbers indicated in the figures as n). Statistical analyses (*n* = 4 or more) were performed using GraphPad Prism 5 or 6. Data are shown as means±SEM (±SD for Fig. 9f–g). For cells and nuclei volumes the two-tailed Mann–Witney test (for non-Gaussian data) or the two-sided unpaired *t* test (for Gaussian data). For the invasive tumour frequency, Chi^2^ test was used. Unpaired *t* test was used to compare human mRNA levels and Two-tailed Mann–Witney test was used for *Drosophil*a mRNA levels. P69 migration tests were analysed with unpaired *t* test.

### Reporting summary

Further information on research design is available in the Nature Research Reporting Summary linked to this article.

## Supplementary information

Reporting Summary

## Data Availability

The human prostate tissues data referenced during the study are available in a public repository from the http://cbio.mskcc.org/cancergenomics/prostate/data/ website. Raw data corresponding to nucleus and cell size, tumour counts, qPCR, human mRNA, eukaryotic cell migration are available as source data. All the other data (imaging) supporting the findings of this study are available within the article from the corresponding author upon reasonable request. A reporting summary for this article is available as a Supplementary Information file.

## Source Data

Source Data
