# Supplementary material for: Sequential Ras/MAPK and PI3K/AKT/mTOR pathways recruitment drives basal extrusion in the prostate-like gland of Drosophila
Source: Nat Commun. 2020 May 8;11:2300. doi: 10.1038/s41467-020-16123-w (PMC7210301; doi:10.1038/s41467-020-16123-w)
Supplement: Supplementary file 1 — Reporting Summary [file 41467_2020_16123_MOESM1_ESM.pdf]

nature research

Corresponding author(s): Cyrille de JOUSSINEAU

Last updated by author(s): Apr 6, 2020

Reporting Summary

Nature Research wishes to improve the reproducibility of the work that we publish. This form provides structure for consistency and transparency in reporting. For further information on Nature Research policies, see Authors & Referees and the Editorial Policy Checklist.

Statistics

For all statistical analyses, confirm that the following items are present in the figure legend, table legend, main text, or Methods section.

n/a	Confirmed	
☐	☒	The exact sample size (*n*) for each experimental group/condition, given as a discrete number and unit of measurement
☐	☒	A statement on whether measurements were taken from distinct samples or whether the same sample was measured repeatedly
☐	☒	The statistical test(s) used AND whether they are one- or two-sided<br>*Only common tests should be described solely by name; describe more complex techniques in the Methods section.*
☒	☐	A description of all covariates tested
☒	☐	A description of any assumptions or corrections, such as tests of normality and adjustment for multiple comparisons
☒	☐	A full description of the statistical parameters including central tendency (e.g. means) or other basic estimates (e.g. regression coefficient) AND variation (e.g. standard deviation) or associated estimates of uncertainty (e.g. confidence intervals)
☐	☒	For null hypothesis testing, the test statistic (e.g. *F*, *t*, *r*) with confidence intervals, effect sizes, degrees of freedom and *P* value noted<br>*Give P values as exact values whenever suitable.*
☒	☐	For Bayesian analysis, information on the choice of priors and Markov chain Monte Carlo settings
☒	☐	For hierarchical and complex designs, identification of the appropriate level for tests and full reporting of outcomes
☒	☐	Estimates of effect sizes (e.g. Cohen's *d*, Pearson's *r*), indicating how they were calculated

Our web collection on statistics for biologists contains articles on many of the points above.

Software and code

Policy information about availability of computer code

Data collection	micrographs came from SP5 and SP8 Leica confocals using Leica application suite X.
Data analysis	Violin plot were made using R package “ggplot2” applying “geomviolin()” function. “geomboxplot()” function was used to add boxplot inside violin. ImageJ: version 2.0.0-rc-69/1.52p for image production, ; Imaris version 9.2 for cell and nucleus sizes; GraphPad Prism 5 and 6 for mRNA and migration statistical tests.

For manuscripts utilizing custom algorithms or software that are central to the research but not yet described in published literature, software must be made available to editors/reviewers. We strongly encourage code deposition in a community repository (e.g. GitHub). See the Nature Research guidelines for submitting code & software for further information.

Data

Policy information about availability of data

All manuscripts must include a data availability statement. This statement should provide the following information, where applicable:

- Accession codes, unique identifiers, or web links for publicly available datasets
- A list of figures that have associated raw data
- A description of any restrictions on data availability

The dataset analysed during the current study is available at http://cbio.mskcc.org/cancergenomics/prostate/data/. Raw data concerning tumour counts, qPCR and migration tests are available as source data files. All other data are available from the corresponding author upon reasonable request.

Field-specific reporting

Please select the one below that is the best fit for your research. If you are not sure, read the appropriate sections before making your selection.

☒ Life sciences ☐ Behavioural & social sciences ☐ Ecological, evolutionary & environmental sciences

For a reference copy of the document with all sections, see nature.com/documents/nr-reporting-summary-flat.pdf

Life sciences study design

All studies must disclose on these points even when the disclosure is negative.

Sample size	No sample size calculation was performed. Typical sample size in the field is 5-20. As chosen animal model displays no ethical concerns, experiments were done on typically more than 50 individuals per condition over a minimum of 4 repeats (see figures for specific numbers)
Data exclusions	no
Replication	Each experiment has been replicated succesfully two or more times.
Randomization	Mostly, organisms were assigned to groups only at the end of the experiment, depending on their genotype. In some cases, flies were raised separatly from eclosion depending on their genotype, for experimental simplicity (possibility to dissect the different genotypes sequentially, and so to minimize the risk of mixed tumor counts).
Blinding	For drosophila, blinding impossible for dissection procedures (invasive tumour counts) as the different genotypes induce different phenotypes (eye color for example). For this reason, mutliple experimentators were assigned to each kind of experiment to test reproducibility. Same procedure was done for cell culture work, with 2 people working together on the experiments. For human data, only one person initially manipulated the data (Yoan Renaud). However, blinding was used in this case as no indication on what could be coherent results were discussed with him before data treatment.

Reporting for specific materials, systems and methods

We require information from authors about some types of materials, experimental systems and methods used in many studies. Here, indicate whether each material, system or method listed is relevant to your study. If you are not sure if a list item applies to your research, read the appropriate section before selecting a response.

Materials & experimental systems

n/a	Involved in the study
☐	☒ Antibodies
☐	☒ Eukaryotic cell lines
☒	☐ Palaeontology
☐	☒ Animals and other organisms
☒	☐ Human research participants
☒	☐ Clinical data

Methods

n/a	Involved in the study
☒	☐ ChIP-seq
☒	☐ Flow cytometry
☒	☐ MRI-based neuroimaging

Antibodies

Antibodies used	We used the following primary antibodies: Mouse Coracle (1:300, #C566.9 DHSB), mouse Fasciclin III (1:400, #7610 DHSB), rat E-Cadherin (1:1000, #DCAD2 DHSB), rabbit P-4E-BP1 (1:200, 2855S Cell Signaling), rabbit P-ERK (1:500, 4370S Cell Signaling), goat GFP (1:1000, #5450 Abcam), mouse GASP (1/5, 2A12 DHSB), mouse MMP1 (1/100, 14A3D2 DHSB ; 1:10 #3A6B4 #3B8D12 #5H7B11 DHSB), rat Spitz (1:100, DHSB), rabbit P-Src (1:500, #44-660G Invitrogen). secondary antibodies coupled to different fluorophores 488 (1:1000, A11055 Invitrogen), Cy5 or Cy3 (1:1000, 711-165-152, 711-175-152, 712-165-153, 712-175-153, 715-165-150, 715-165-151, 715-175-150, 715-175-151, Jackson Immunology)
Validation	All the antibodies are commercialy available, validated by the manufacturers for drosophila use and immunohistochemistery, and already published in scientific papers (examples below for primary antibodies).<br>P-4E-BP1, Fasciclin 3, Coracle and GFP antibodies along with phalloïdin conjugates were already published in Vachias et al, 2014, DOI:https://doi.org/10.1016/j.celrep.2014.09.035<br>P-ERK antibody : Xu et al, eLife 2017, DOI: 10.7554/eLife.22441.001<br>E-Cadherin antibody : Ito et al, 2014 : DOI: 10.1016/j.bbrc.2014.08.015<br>GASP antibody : Tiklova et al, 2013 : DOI: 10.1371/journal.pone.0067415<br>MMP1 antibody : Sauerwald et al, Elife. 2019. doi: 10.7554/eLife.48857.<br>Spitz antibody : Schweitzer et al, Genes Dev 1995, DOI:10.1101/gad.9.12.1518. For immunohistochemistry purpose, we validated antibody on clones expressing an RNAi against Spitz.<br>P-Src antibody : Bangi et al, Nat Commun 2016, doi: 10.1038/ncomms13615

Eukaryotic cell lines

Policy information about cell lines

Cell line source(s)	P69 cell line was obtained from Bost laboratory (C3M laboratory, Nice, France) through collaborative exchange.
Authentication	Morphologic characteristics and very low growth rate specific to non tumoral cells. Source batch published previously in Ben Sarha et al, Oncogene 2008, doi: 10.1038/sj.onc.1211024.
Mycoplasma contamination	Tested, negative.
Commonly misidentified lines (See ICLAC register)	None. Not amongst misidentified cell lines (source : iclac.org, version 10).

Animals and other organisms

Policy information about studies involving animals; ARRIVE guidelines recommended for reporting animal research

Laboratory animals	Drosophila melagonaster, W1118, males, aged 3-4 days.
Wild animals	No wild animals were collected in the study
Field-collected samples	No field-collected samples were used in the study
Ethics oversight	Not applicable to mentioned animal model

Note that full information on the approval of the study protocol must also be provided in the manuscript.
